# Improved Synthesis of Deoxyadenosine Triphosphate by *Saccharomyces cerevisiae* Using an Efficient ATP Regeneration System: Optimization of Response Surface Analysis

**DOI:** 10.3390/molecules28104029

**Published:** 2023-05-11

**Authors:** Jian Xiong, Hanghang Xu, Qi Wang, Wenyuan Sun

**Affiliations:** School of Chemistry and Chemical Engineering, Henan University of Science and Technology, Luoyang 471023, China

**Keywords:** deoxyadenosine triphosphate, whole cell catalysis, *Saccharomyces cerevisiae*, optimization, energy regeneration system, response surface analysis

## Abstract

Deoxyadenosine triphosphate (dATP) is an important biochemical molecule. In this paper, the synthesis of dATP from deoxyadenosine monophosphate (dAMP), catalyzed by *Saccharomyces cerevisiae,* was studied. By adding chemical effectors, an efficient ATP regeneration and coupling system was constructed to achieve efficient synthesis of dATP. Factorial and response surface designs were used to optimize process conditions. Optimal reaction conditions were as follows: dAMP 1.40 g/L, glucose 40.97 g/L, MgCl_2_·6H_2_O 4.00 g/L, KCl 2.00 g/L, NaH_2_PO_4_ 31.20 g/L, yeast 300.00 g/L, ammonium chloride 0.67 g/L, acetaldehyde 11.64 mL/L, pH 7.0, temperature 29.6 °C. Under these conditions, the substrate conversion was 93.80% and the concentration of dATP in the reaction system was 2.10 g/L, which was 63.10% higher than before optimization, and the concentration of product was 4 times higher than before optimization. The effects of glucose, acetaldehyde, and temperature on the accumulation of dATP were analyzed.

## 1. Introduction

Deoxyadenosine triphosphate (dATP) is an essential precursor material for synthetic DNA [1,2] and is widely used in genetic engineering, molecular biology, life sciences, genetic medicine, etc. [3,4,5]. Currently, dATP is mainly produced commercially by chemical methods. The reaction is carried out with tributylammonium salt and orthophosphoric acid corresponding to deoxyadenosine monophosphate (dAMP) as substrates, dicyclohexylcarbodiimide (DCC) as the catalyst, and organic solvents such as pyridine or dimethylformamide (DMF). The yield of dATP produced by the chemical method is 40~80% [6,7], generates great environmental pollution and its cost is relatively high after reaction and purifi-cation [8]. With the development of biotechnology, the biosynthesis of deoxynucleoside triphosphate is more advantageous than the chemical method [9,10]. There are usually two pathways for the biosynthesis of dATP. The first pathway consists of two−step phosphorylation reaction, namely dAMP and dADP phosphorylation. That is, dAMP is catalyzed by deoxynucleoside monophos-phate kinase to produce dADP, and dADP is further catalyzed by pyruvate kinase to synthesize DATP. Both of phosphorylation processes require the participation of ATP [11,12,13,14]. However, this pathway often requires the addition of two enzymes and ATP as reaction raw materials, and the reaction cost is very high; furthermore, the yield of dATP is still low, only about 60% [15,16,17]. Ding et al. constructed intact nucleotide kinases and acetate kinase cells with the N-terminus of the ice nucleation protein (INP-N-NMKases and INP-N-ACKase cells) using cell surface display technology. The cells were used to synthesize dATP, and the yield can reach more than 90% [18]. The second pathway is to use nucleotide reductase for to remove the hydroxyl groups on the adenosine triphosphate substrate, thus generating the corresponding deoxyadenosine triphosphate. However, this enzyme has poor stability and is not suitable for industrial production [19].

**Figure 1:**
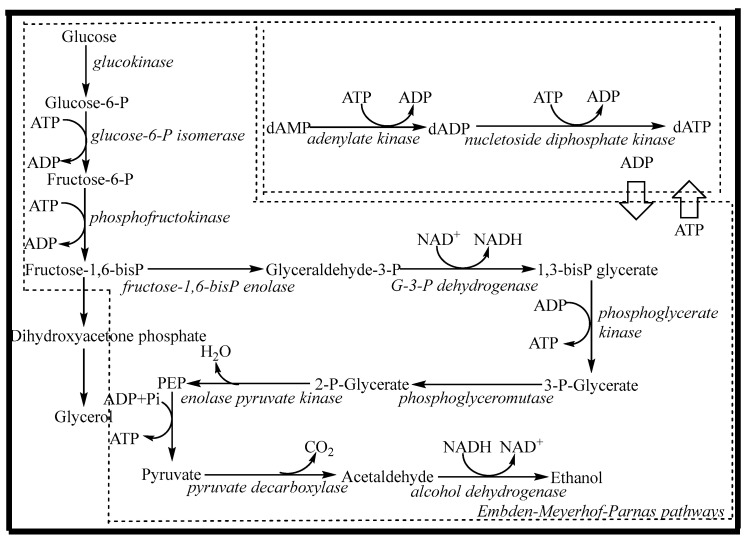
The biosynthesis pathway of dATP by *Saccharomyces cerevisiae*.

In order to overcome the above problems, the whole cell can be considered for dATP biosynthesis. As shown in Figure 1, the synthetic pathway mainly consists of two parts: the energy (ATP) regeneration pathway (glycolysis pathway, or EMP pathway) and the dATP synthesis pathway. In whole cells, the glycolysis pathway exists as an ATP regeneration system that can break down one molecule of glucose into two molecules of ethanol and produce two molecules of ATP. The dATP synthesis system requires two molecules of ATP for phosphorylation to produce one molecule of dATP. By coupling ATP regeneration and utilization with dAMP phosphorylation, dATP can be effectively synthesized. Therefore, the regeneration and coupling of ATP are prerequisites for the efficient synthesis of dATP. At present, the efficiency of ATP generated by sugar through the EMP pathway is very low, which can only maintain the general metabolism of cells. To increase the flux of the EMP pathway and overexpress the phosphorylation level of substrate, the main approaches are the use of genetic engineering technology or changing the metabolic flux by using chemical effector substances [20,21,22]. The latter is more convenient and easier to achieve. The EMP pathway is a multi-enzyme catalytic system in which some enzymes, especially allosteric enzymes such as glucokinase (GK), phosphofructokinase (PFK), and pyruvate kinase (PK), are the key enzymes. Each of these enzymes has its own regulatory factors, in which magnesium ions and potassium ions have regulatory effects on one or more of the above enzymes [23,24]. An appropriate concentration of ammonium ions can increase the activity of phosphofructokinase, allowing the energy in glucose to be stored in the form of fructose-1,6-diphosphate. During the subsequent phosphorylation of dAMP, sufficient energy was provided for substrate-level phosphorylation, which was beneficial for product synthesis [25]. In the EMP pathway, NAD^+^ participates in the reaction as a proton-transporting coenzyme and reduces itself to NADH. This reaction is catalyzed by glyceraldehyde-3-phosphate dehydrogenase and is the only redox reaction in the EMP pathway. However, the intracellular redox cofactor pool is fixed, and there is a dynamic balance between oxidation and reduction. For glycolysis to continue, the reduced NADH must be reoxidized to the oxidized form (NAD^+^) through the regeneration pathway. With a lack of oxygen or an oxidant factor, NAD^+^ will regenerate via the ethanol pathway, using acetaldehyde as an electron acceptor [26]. Therefore, under the regulation of chemical effector substances (Mg^2+^, K^+^, NH_4_^+^, and acetaldehyde), the regeneration rate of ATP can be increased, and when the rate matches that of the dATP synthesis system, efficient biosynthesis of dATP can be realized.

In this paper, a new technique for the wholecell biosynthesis of dATP by *Saccharomyces cerevisiae* is described. Magnesium chloride hexahydrate (MgCl_2_·6H_2_O), potassium chloride (KCl), ammonium chloride (NH_4_Cl), and acetaldehyde were selected as effectors to regulate the metabolic flux of the EMP pathway and establish an efficient ATP regeneration and coupling system. Response surface analysis (RSA) and factorial experiments were used to optimize the synthesis of dATP from dAMP catalyzed by *Saccharomyces cerevisiae*. As a mathematical analysis method, RSA is often used to explore the influence of multiple parameters on dependent variables. RSA can also be used to predict the value of the dependent variable for specific parameters or obtain the optimal operating parameters based on the expected value of the dependent variable. A central composite rotatable design (CCRD)-type RSA, an ideal tool for process optimization [27,28], was used to optimize the synthesizing process. The effects of glucose, acetaldehyde, and temperature on the accumulation of dATP were also determined. Yeast whole cell catalysis technology can overcome the problems of low substrate utilization efficiency, low product yield, and high production costs in the synthesis process. At the same time, compared to enzymatic methods, the use of whole cells results in better enzyme stability, greater adaptability to organic solvents, and easier in situ regeneration of energy and coenzymes.

## 2. Results and Discussion

### 2.1. Preliminary Synthesis of dATP

**Figure 2:**
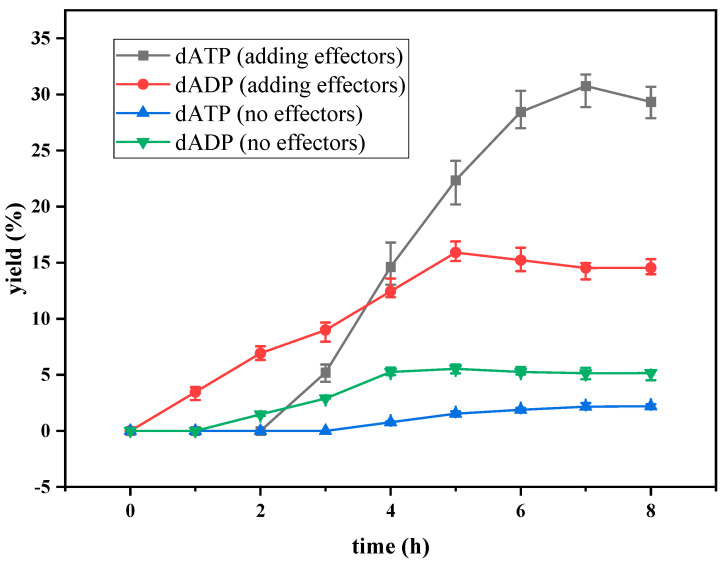
The yield of dATP and dADP in preliminary experiments.

The curves of the yield of dATP and dADP versus time with and without the addition of effectors are shown in Figure 2. When no effector was added to the reaction system, the accumulation of dATP was detected after 4 h of reaction. The reaction efficiency was very low, and the yield of dATP was only 2.20% after 8 h of reaction. dADP was detectable at 2 h and remained basically the same after 4 h of reaction. The yield of dADP was 5.16% at 8 h. The results indicated that ATP provided by the EMP pathway in yeast cells could not satisfy dATP synthesis, and only a small amount of substrate dAMP could be converted to dADP and dATP without effectors. When the effectors were added to the reaction system, dATP could be detected after 2 h of reaction. The yield of dATP increased linearly from 2 h to 6 h. The maximum yield of dATP was 30.7% at 7 h. According to the change in dADP, the yield increased gradually in the first 5 h, but the increasing range was very slow. After 5 h, the yield of dADP remained basically stable at about 14%. The addition of the effectors greatly improved the regeneration efficiency of ATP in the EMP pathway, which was beneficial to the phosphorylation level of dAMP. Through the regeneration and coupling of ATP, the effective synthesis of dATP was realized. However, under these conditions, the yield of dATP was still low, so it was necessary to optimize the synthesis conditions to improve the yield and efficiency of the synthesis of dATP.

### 2.2. Fractional Factorial Design to Identify Key Influences

Based on preliminary experiments and literature [29], the following ten factors were determined for the experimental design: dAMP, glucose, MgCl_2_·6H_2_O, KCl, NaH_2_PO_4_, yeast, NH_4_Cl, acetaldehyde, pH, and temperature. Parameter values and coding levels are shown in Table 1. The experimental data and the predicted values are shown in Table 2, which was analyzed with Design Expert version 13. The function of the coded levels of all factors was obtained:

Y = 36.5 − 1.05x_1_ + 10.86x_2_ + 0.43x_3_ − 1.48x_4_ + 5.75x_5_ + 1.93x_6_ + 1.38x_7_ − 13.61x_8_ + 1.49x_9_ + 7.84x_10_ (1)

The determination coefficient (R^2^) for the linear regression model of dATP production was 0.905, indicating that the results of this analysis are -reliable. Furthermore, from the results of the analysis (Table 3), it is seen that glucose, acetaldehyde, and temperature had a significant effect on dATP production (*p* ≤ 0.05). When glucose and temperature were near high-level values and acetaldehyde was near a low-level value, it was beneficial to improve the dATP yield. According to the trends of the three factors, other factors with less influence were taken as fixed values (the average of level −1 and level 1) as follows: x_1_ 1.40, x_3_ 4.00, x_4_ 2.00, x_5_ 31.20, x_6_ 300.00, x_7_ 0.67, x_9_ 7.0. Response surface design with glucose, acetaldehyde, and temperature as variables.

### 2.3. Central Composite Design and Response Surface Methodology

According to the results of the fractional factorial design, glucose, acetaldehyde, and temperature had significant effects, and a response surface design with three factors at five levels was used to determine the optimal levels. The experimental design and results are shown in Table 4 and Table 5. The results of the regression analysis are shown in Table 6. The fitted second-order polynomial had the following form:

Y = 91.19 + 0.97x_1_ − 22.03x_1_^2^ − 6.02x_2_ − 17.65x_2_^2^ − 5.05x_3_ − 24.58x_3_^2^ − 1.27x_1_x_2_ − 6.53x_1_x_3_ + 15.50x_2_x_3_ (2)

where Y is the predicted response, and x_1_, x_2_, and x_3_ are coded values of glucose, acetaldehyde, and temperature concentrations, respectively.

**Figure 3:**
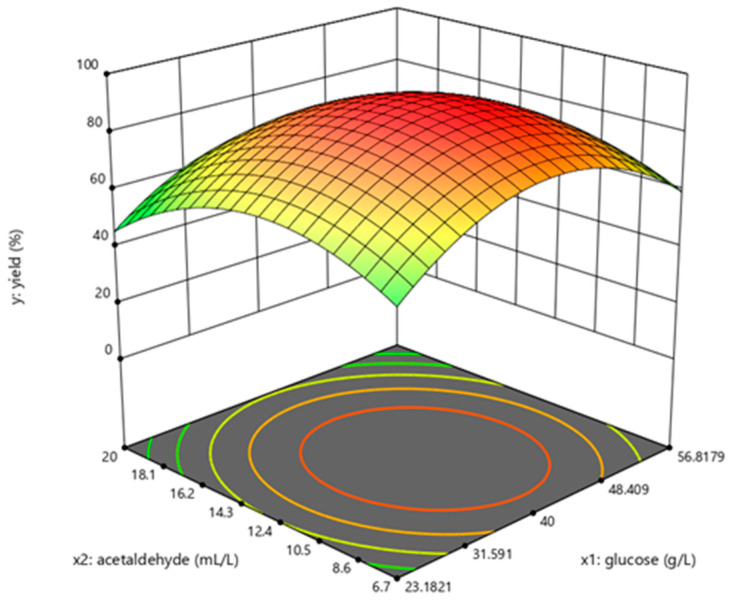
Response surface curve for dATP synthesis by *Saccharomyces cerevisiae* when temperature was maintained at 30 °C.

**Figure 4:**
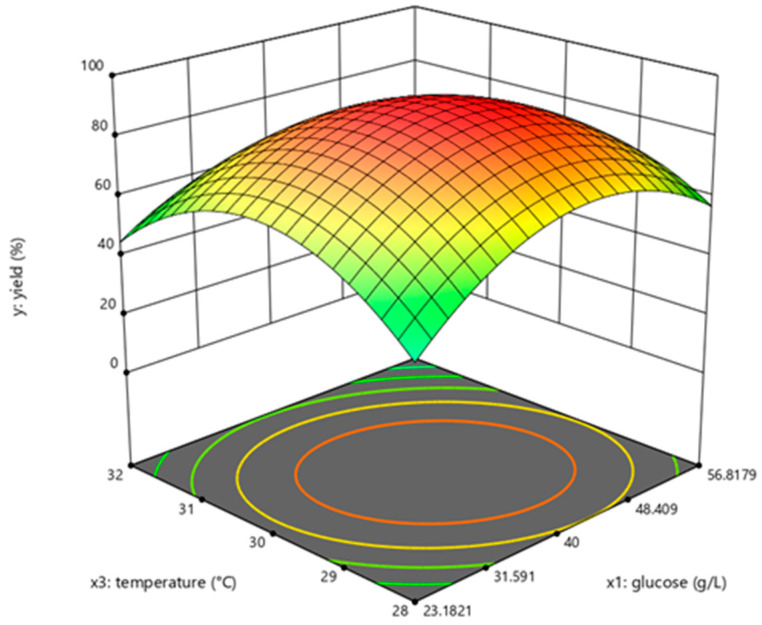
Response surface curve for dATP synthesis by *Saccharomyces cerevisiae* when acetaldehyde was maintained at 13.4 mL/L.

**Figure 5:**
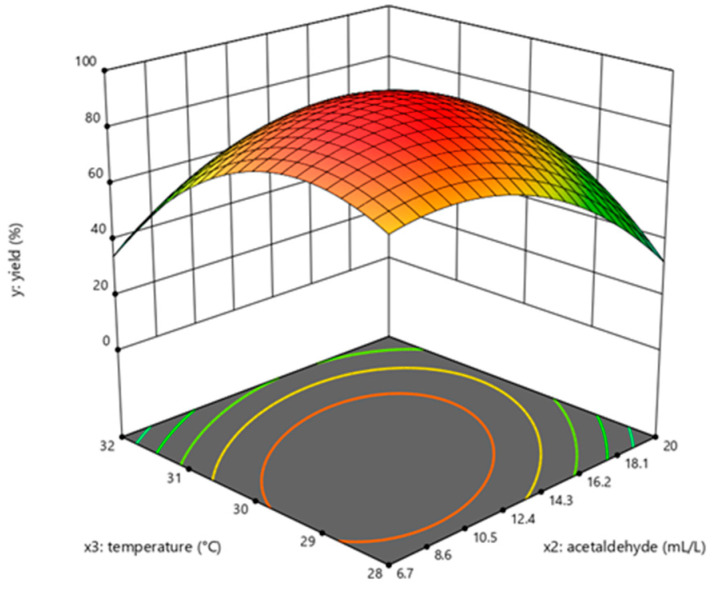
Response surface curve for dATP synthesis by *Saccharomyces cerevisiae* when glucose was maintained at 40 g/L.

**Figure 6:**
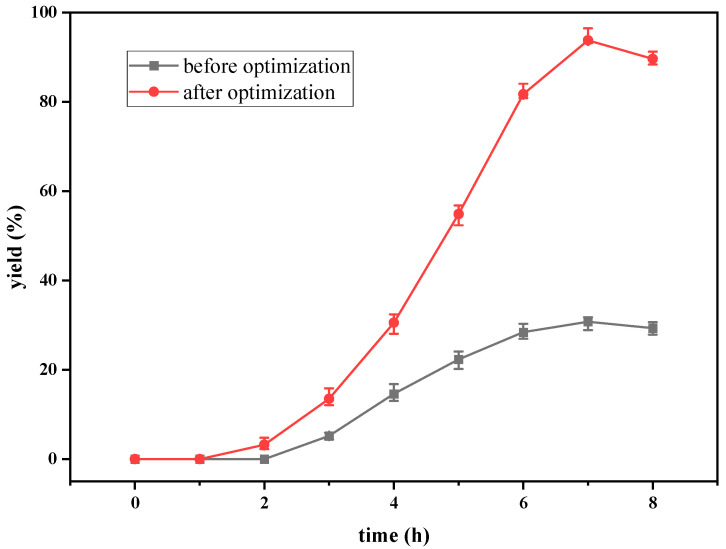
Comparison chart of dATP yield before and after optimization.

The determination coefficient (R^2^) for the equation was 0.897. First-order partial derivatives of Y for x_1_, x_2_, and x_3_ were obtained, and the values were set to zero, respectively. Combining the above equations to solve them, the values of x_1_, x_2_, and x_3_ were 40.97, 11.64, and 29.62, respectively. The model predicted a maximum response of 92.47% under these conditions. Also, the 3D response surface curves were then plotted to present the effect of two factors while the other factor was held at zero (Figure 3, Figure 4 and Figure 5). The optimized reaction conditions were obtained by combining the previous values of other parameters: dAMP was 1.40 g/L, glucose was 40.97 g/L, MgCl_2_·6H_2_O was 4.00 g/L, KCl was 2.00 g/L, NaH_2_PO_4_ was 31.20 g/L, yeast was 300.00 g/L, NH_4_Cl was 0.67 g/L, acetaldehyde was 11.64 mL/L, pH was 7.0, and the temperature was 29.6 °C. The experiments were conducted under these conditions, and the final concentration of dATP was 2.10 g/L with an actual yield of 93.80% (Figure 6). The concentration of dATP was four times higher than the pre-optimization concentration (0.52 g/L). The yield of dATP increased by 63.10% compared to the starting yield of 30.70%. The above results indicate that the dATP yield and concentration were greatly improved after optimization, which reduced the unit product cost. It also indicates that the optimization using FFD and RSA is more credible and effective.

### 2.4. Effect of Glucose on dATP Synthesis

The phosphorylation of dAMP requires the glycolysis pathway to provide ATP, and glucose is the only energy donor in this conversion system. If the glucose concentration is too low, the reaction energy is insufficient, and not enough ATP can be produced, resulting in a decrease in the conversion rate. If the glucose concentration is too high, a large amount of ATP is required for glucose phosphorylation at the initial stage of the reaction, and the sugar phosphate compounds (glucose 6-phosphate, fructose 6-phosphate, and fructose 1,6-diphosphate) accumulate [30], while the ATP used for dAMP phosphorylation is insufficient [31]. In addition, excessive glucose 6-phosphate has feedback inhibition on hexokinase, which slows glucose phosphorylation and then affects the rate of glycolysis, thus reducing the conversion rate. Through optimization, 40.97 g/L glucose was selected as the optimal concentration.

### 2.5. Effect of Acetaldehyde on dATP Synthesis

There are two ways to regenerate NAD^+^ in yeast cells [32]. In the case of high dissolved oxygen, NAD^+^ is regenerated via the electron transport chain (ETC) of mitochondria, with oxygen acting as the final electron acceptor. Under low-dissolved oxygen or anaerobic conditions, NAD^+^ can be regenerated by using the ethanol pathway and acetaldehyde as an exogenous electron acceptor. Exogenous acetaldehyde can oxidize NADH to NAD^+^ under the action of ethanol dehydrogenase, while acetaldehyde can be reduced to the same amount of ethanol, which accelerates the regeneration of NAD^+^, maintains the ratio NADH/NAD^+^ in cells, and restores the redox balance of cells [33]. Acetaldehyde can transfer the NADH oxidation pathway to the ethanol fermentation pathway, thereby increasing the flux of the glycolysis pathway and promoting the phosphorylation of dAMP to dATP. Intracellular NADH excess inhibits the reaction catalyzed by 3-phosphate glyceraldehyde dehydrogenase, which oxidizes 3-phosphate glyceraldehyde to 1,3-diphosphoglyceric acid. This reaction is the first step of the sugar fermentation interpretation reaction and is also the metabolic entry port for substrate-level phosphorylation. The addition of acetaldehyde can increase the concentration of NAD^+^, reverse this effect, and then accelerate the regeneration of ATP [34,35]. With an increase in the concentration of acetaldehyde, the yield of dATP increased. Acetaldehyde, as an exogenous electron acceptor, also has a certain cytotoxicity, which can form complexes with proteins [36], hinder protein function, and lead to a decrease in dATP yield. After optimization, 11.64 mL/L of acetaldehyde was selected as the final concentration.

### 2.6. Effect of Temperature on dATP Synthesis

Temperature has two effects on the enzymatic reaction rate. First, when the temperature increases, the reaction speed increases, which is similar to the general chemical reaction. On the other hand, when the temperature rises to a certain extent, the enzyme begins to denature. This means that the reaction speed of the enzyme is reduced due to the decrease in active enzymes. The optimum temperature of the enzymatic reaction is the result of the equilibrium between these two processes. Below the optimum temperature, the former effect plays a dominant role; that is, the conversion rate increases with the increase in temperature. When the temperature is higher than the optimal temperature, the latter effect plays a dominant role, so the enzyme activity is rapidly lost, the reaction rate decreases rapidly, and the yield of dATP decreases. By optimization, 29.6 °C is selected as the experimental temperature.

## 3. Materials and Methods

### 3.1. Strain and Medium

*Saccharomyces cerevisiae* As2.398, preserved in the laboratory of Henan University of Science and Technology, was used for the production of dATP from dAMP in this study. The growth medium contained 5% glucose, 0.5% peptone, 0.2% yeast extract, 0.2% NH_4_H_2_PO_4_, 0.1% MgSO_4_·7H_2_O, 0.2% NH_4_SO_4_, and 0.2% KH_2_PO_4_ at an initial pH of 5.8. The culture medium was kept at 30 °C for 72 h.

### 3.2. Preparation of Cells

The yeast cells were harvested aseptically using the vacuum filtration method at 4 °C and washed twice with deionized water. The wet cells were frozen and stored at −20 °C.

### 3.3. dATP Synthesis Process

Reaction components such as yeast, glucose, and inorganic salts were accurately weighed into a 500 mL Erlenmeyer flask and diluted to 300 mL with distilled water. The reactions were carried out in an air-insulated condition with shaking in a thermostat-controlled water bath at 120 r/min for 7 h. All experiments were carried out in triplicate.

The reaction conditions without the addition of effectors were as follows: dAMP, 1.40 g/L; glucose, 30.00 g/L; NaH_2_PO_4_, 31.20 g/L; yeast cell, 250.00 g/L; pH, 7.0; temperature, 30.0 °C.

The reaction conditions with the addition of effectors were as follows: dAMP, 1.40 g/L; glucose, 30.00 g/L; MgCl_2_·6H_2_O, 2.00 g/L; KCl, 1.33 g/L; NaH_2_PO_4_, 31.20 g/L; yeast cell, 250.00 g/L; NH_4_Cl, 1.00 g/L; acetaldehyde, 6.70 mL/L; pH, 7.0; temperature, 30.0 °C. 

The precise compositions and reaction conditions of optimized experiments are described in the Section 2. 

### 3.4. Analytic Method

The reaction aliquots were centrifuged at 10,000× *g* for 10 min, and the supernatant was used to determine dAMP, dADP, and dATP. High-performance liquid chromatography (HPLC, Agilent 1100 system with UV detector) was performed using a Lichrospher C18 column (4.6 mm × 300 mm, 5 µm), methanol 0.05 mol/L of dipotassium phosphate solution (3:97, *v*/*v*) as the mobile phase, and a flow rate of 1.0 mL/min at room temperature. The detection wavelength was 254 nm. The retention times for dAMP, dADP, and dATP were 6.118 min, 8.871 min, and 14.079 min, respectively.

### 3.5. Fractional Factorial Design (FFD)

Ten factors, such as dAMP, glucose, MgCl_2_·6H_2_O, KCl, NaH_2_PO_4_, yeast, ammonium chloride, acetaldehyde, pH, and temperature, were selected as the investigation objectives. If the whole factor design was used, there would be 10 factors and 2 levels of the 2^10^ designs, which required many experiments and was difficult to realize. Compared with the full-factor design, FFD can greatly reduce the number of experiments without losing the main information and can estimate the main effect and partial interaction of factors. In the experimental system of this study, a FFD of ten factors and two levels is selected. Each has two levels and is coded by (−1, +1) (Table 1). The experiments are carried out according to the corresponding experimental table, with a total of 16 experiments (Table 2). Based on the analysis of the results of the factorial design, the relatively important influencing factors are determined.

The first-order model used to fit the results of fractional factorial design was represented as:

(3) $$Y=\beta_{0}+{\sum \beta }_{i}x_{i}$$

where Y is the predicted response; β_0_ is the intercept; β_i_ is the linear coefficient; and x_i_ is the coded independent factor.

### 3.6. Response Surface Analysis

Response surface analysis (RSA) is a mathematical and statistical method for investigating the optimal conditions corresponding to the maximum response value of a factor interaction in a multifactorial system [37,38,39]. Although based on the principles of orthogonal design, it is more effective than the previously promoted “orthogonal experimental method”.

In this study, the three independent factors were studied in one block, and a set of 16 experiments was performed in a random order with six axial points, eight factorial points, and two center points. After regression fitting, the influence of each experimental factor on the response value can be expressed by the following functions:

(4) $$Y=\beta_{0}+\sum \beta_{i}x_{i}+\sum \beta_{ii}x_{i}^{2}+\sum \beta_{ij}x_{i}x_{j}$$

where Y is the predicted response, β_0_ is the intercept, x_i_ and x_j_ are the coded independent factors, β_i_ is the linear coefficient, β_ii_ is the quadratic coefficient, and β_ij_ is the interaction coefficient.

Design Expert version 13 (STATEASE Inc., Minneapolis, MN, USA) was used for experimental designs and regression analysis of the experimental data obtained.

## 4. Conclusions

In this study, a whole cell method for the biocatalytic synthesis of dATP in *Saccharomyces cerevisiae* has been designed, establishing a regeneration and energetic coupling system capable of efficient dATP biosynthesis. A multinomial quadratic mathematical model of the dATP biosynthesis was established by the statistical method. Through the factorial experiments and the optimization of the response surface, optimal conditions affecting the biosynthesis of dATP by *Saccharomyces cerevisiae* were obtained, and the biosynthesis of dATP was predicted using the model equation. Under optimal conditions, the yield and concentration of dATP reached 93.80% and 2.10 g/L, respectively. The yield was 63.10% greater than the level before optimization, and the concentration increased nearly fourfold. As an important substrate for DNA synthesis, the establishment of the dATP biosynthesis system provides a powerful means for gene cloning, protein engineering, biomedical research, and development and is of far-reaching significance. Compared with other methods reported in the literature, the method in this study does not require complex genetically engineered bacteria construction and protein expression and has the advantages of simple operation, low cost, and high efficiency. Moreover, this method would allow the production of other active substances that need ATP as energy, such as S-adenosyl-L-methionine (SAM), glutathione, penicillin and its derivatives, poly-amino acids, and polysaccharides, which have a broad application prospect.

## Figures and Tables

**Table 1:** The values of the variables and the coding level.

Coding Level	x_1_	x_2_	x_3_	x_4_	x_5_	x_6_	x_7_	x_8_	x_9_	x_10_
	dAMP (g/L)	Glucose (g/L)	MgCl_2_·6H_2_O (g/L)	KCl (g/L)	NaH_2_PO_4_ (g/L)	Yeast (g/L)	NH_4_Cl (g/L)	Acetaldehyde (mL/L)	pH	T (°C)
−1	0.80	30.00	1.00	1.33	26.00	250.00	0.33	6.70	6.5	28.0
+1	2.00	50.00	7.00	2.67	36.40	350.00	1.00	20.00	7.5	32.0

**Table 2:** Experimental design and results of fractional factorial design.

Run	Factors										Y (%)	Y′ (%)
	x_1_	x_2_	x_3_	x_4_	x_5_	x_6_	x_7_	x_8_	x_9_	x_10_		
1	−1	−1	−1	−1	−1	−1	−1	−1	1	1	41.68	41.25
2	1	−1	−1	−1	1	−1	1	1	−1	−1	9.87	7.54
3	−1	1	−1	−1	1	1	−1	1	−1	−1	33.71	32.48
4	1	1	−1	−1	−1	1	1	−1	1	1	70.64	67.50
5	−1	−1	1	−1	1	1	1	−1	−1	1	44.93	57.27
6	1	−1	1	−1	−1	1	−1	1	1	−1	5.90	0.98
7	−1	1	1	−1	−1	−1	1	1	1	−1	15.22	23.71
8	1	1	1	−1	1	−1	−1	−1	−1	1	79.03	70.25
9	−1	−1	−1	1	−1	1	1	1	−1	1	23.53	14.75
10	1	−1	−1	1	1	1	−1	−1	1	−1	27.40	35.89
11	−1	1	−1	1	1	−1	1	−1	1	−1	63.54	58.62
12	1	1	−1	1	−1	−1	−1	1	−1	1	15.83	27.72
13	−1	−1	1	1	1	−1	−1	1	1	1	26.59	23.45
14	1	−1	1	1	−1	−1	1	−1	−1	−1	22.39	21.16
15	−1	1	1	1	−1	1	−1	−1	−1	−1	48.42	46.09
16	1	1	1	1	1	1	1	1	1	1	50.13	49.70

Y: experimental data; Y′: predicted values.

**Table 3:** Results of the regression analysis of the fractional factorial design.

Term	Regression Analysis		
	Coefficient	F-Value	*p*-Value
x_1_	−1.05	0.1299	0.7332
x_2_	10.86	13.7700	0.0138
x_3_	0.43	0.0215	0.8893
x_4_	−1.48	0.2539	0.6357
x_5_	5.75	3.8600	0.1066
x_6_	1.93	0.4370	0.5378
x_7_	1.38	0.2235	0.6563
x_8_	−13.61	21.6100	0.0056
x_9_	1.49	0.2591	0.6324
x_10_	7.84	7.1800	0.0439

**Table 4:** Levels of the factors tested in the central composite design.

Factors	Levels of Factors				
	−1.618	−1	0	1	1.618
x_1_ (glucose, g/L)	23.2	30	40	50	56.8
x_2_ (acetaldehyde, mL/L)	2.2	6.7	13.4	20	24.5
x_3_ (temperature, °C)	26.6	28	30	32	33.4

**Table 5:** Experimental design and results of the RSM.

Run	x_1_	x_2_	x_3_	Y (%)	Y′ (%)
1	1	−1	1	23.24	24.09
2	0	0	0	90.18	91.19
3	−1	−1	1	19.55	29.18
4	−1	1	−1	11.33	21.00
5	0	0	0	89.64	91.19
6	0	−1.618	0	54.65	51.38
7	−1.618	0	0	86.46	68.19
8	−1	−1	−1	56.03	62.52
9	0	0	1.618	24.73	13.17
10	0	1.618	0	42.74	31.13
11	−1	1	1	36.19	49.65
12	1	1	−1	27.52	28.40
13	0	0	−1.618	33.47	30.16
14	1	−1	−1	75.90	72.95
15	1.618	0	0	66.73	70.13
16	1	1	1	37.50	41.53

Y: experimental data; Y′: predicted values.

**Table 6:** Regression results of the central composite design.

Term	Regression Analysis		
	Coefficient	F-Value	*p*-Value
x_1_	0.97	0.0254	0.8787
x_2_	−6.02	2.7600	0.1476
x_3_	−5.05	1.9500	0.2125
x_1_^2^	−22.03	0.0256	0.8781
x_2_^2^	−17.65	0.6731	0.4433
x_3_^2^	−24.58	10.7200	0.0169
x_1_x_2_	−1.27	3.1400	0.1270
x_1_x_3_	−6.53	16.1100	0.0070
x_2_x_3_	15.50	31.2400	0.0014

## Data Availability

Raw data are available on request.

## Sample Availability

Samples of the compounds are available from the authors.
